# ARM4CH: A Methodology for Autonomous Reality Modelling for Cultural Heritage

**DOI:** 10.3390/s24154950

**Published:** 2024-07-30

**Authors:** Nikolaos Giakoumidis, Christos-Nikolaos Anagnostopoulos

**Affiliations:** 1 KINESIS Lab, Core Technology Platforms, New York University Abu Dhabi, Abu Dhabi P.O. Box 129188, United Arab Emirates; giakoumidis@nyu.edu; 2 Intelligent Systems Lab, Cultural Technology and Communication, University of the Aegean, 811 00 Mitilini, Greece

**Keywords:** reality modeling, autonomous robots, terrestrial laser scanning, LiDAR, UAV, Next Best View

## Abstract

Nowadays, the use of advanced sensors, such as terrestrial, mobile 3D scanners and photogrammetric imaging, has become the prevalent practice for 3D Reality Modeling (RM) and the digitization of large-scale monuments of Cultural Heritage (CH). In practice, this process is heavily related to the expertise of the surveying team handling the laborious planning and time-consuming execution of the 3D scanning process tailored to each site’s specific requirements and constraints. To minimize human intervention, this paper proposes a novel methodology for autonomous 3D Reality Modeling of CH monuments by employing autonomous robotic agents equipped with the appropriate sensors. These autonomous robotic agents are able to carry out the 3D RM process in a systematic, repeatable, and accurate approach. The outcomes of this automated process may also find applications in digital twin platforms, facilitating secure monitoring and the management of cultural heritage sites and spaces, in both indoor and outdoor environments. The main purpose of this paper is the initial release of an Industry 4.0-based methodology for reality modeling and the survey of cultural spaces in the scientific community, which will be evaluated in real-life scenarios in future research.

## 1. Introduction

In recent years, Reality Modeling (RM) technologies, including cutting-edge sensors and systems such as LiDAR-based 3D scanners, drones, digital twins, augmented reality (AR), and virtual reality (VR), have become increasingly significant in the field of Cultural Heritage (CH) modeling, recording, and management [1,2,3,4,5]. However, the RM of CH remains a significant challenge for surveyors, as the 3D modeling process is largely manual, labor-intensive, and time-consuming. The scanning path and sensor positioning are predominantly reliant on the surveyor’s experience, intuition, and perception, as there is currently no standardized automatic procedure [6]. The complexity is compounded by the natural environment surrounding CH sites, the morphological intricacies, and the vulnerability of the monuments. Specifically, to acquire a complete 3D reality model of a large-scale cultural space, multiple manual terrestrial 3D scans (TLS) and aerial surveys with unmanned aerial vehicles (UAVs) are required [1]. This manual approach heavily depends on the operator’s expertise to determine the scanning path and identify the optimal scanner positions, a task known in the literature as the Next Best View (NBV) problem. Consequently, optimizing the NBV to efficiently capture large-scale, complex sites or monuments in dynamic environments (e.g., due to growing or changing vegetation) is crucial to reduce surveying time and enhance data quality. Despite its importance, the NBV problem has not been adequately addressed in terms of efficiency and optimality in existing literature. As a result, the surveying process often takes longer than necessary, with redundant overlaps and additional positions planned as a precaution.

To address these challenges, this paper proposes a technological platform for autonomous 3D Reality Modeling and scanning. The goal is to develop a comprehensive, autonomous, systematic, and optimized 3D scanning procedure that accelerates the overall RM process and enhances data quality. To achieve this, two scientific pillars are essential: (a) a framework consisting of Robotic Agents (RAs) equipped with RM sensors that can navigate and operate autonomously and (b) a methodology to identify the optimal positions and trajectories for scanning, applicable to both terrestrial and aerial surveys, which maximizes area coverage and minimizes the number of scanning positions required (addressing the NBV problem). This approach aims to streamline the 3D RM process, ensuring efficient and high-quality data acquisition for cultural heritage sites.

The contributions of the proposed methodology (ARM4CH) are manifold and may be summarized in the following bullet points with more details available in the table in Section 4:

- Non-invasive and autonomous survey and inspection;
- Scanning operation for hard-to-reach, complex or dangerous areas;
- Reduction of labor costs and time-consuming scanning processes;
- Versatility and an increase in data precision;
- Consistency and optimization of measurements and data acquisition;
- Scanning and survey reproducibility;
- Regular monitoring of a CH site;
- Long-Term Monument Preservation and Management, a fostering of the Digital Twin concept.

## 2. Robotic Agents and 3D Scanning: A Brief Overview

Three-dimensional scanning using mobile robots has been already applied in recent years, especially in the field of construction, in which 3D scanning and monitoring is required on a regular basis. In most of these scenarios, the robots follow a predefined path or rely on exploration algorithms [7]. Recent years have seen extraordinary progress in the field of robotics, fueled by several key developments. The adoption of advanced control algorithms, such as Model Predictive Control and Deep Reinforcement Learning, advanced the creation of diverse locomotion mobile robots, including bio-inspired quadrupeds capable of navigating through challenging terrains [8,9]. Moreover, the emergence of advanced perception sensors like Depth Cameras, LiDAR, and Global Navigation Satellite Systems (GNSS) and torque-force sensors have revolutionized data acquisition, enabling the capture of extensive, detailed information that offers accurate and comprehensive insights for both environmental conditions and the robots’ positions. The integration of artificial intelligence (AI) and machine learning algorithms into robotic systems [10] significantly enhances their autonomy, adaptability, and decision-making capabilities. This is further supported by increased processing power, which facilitates the application of sophisticated perception and AI algorithms directly on the robots, enhancing their efficiency and responsiveness. Moreover, hardware advancements, including batteries with higher energy density [11], more powerful computing units, and more efficient motors, have further advanced the capabilities of robotic systems.

Quadrupedal robots have been utilized for 3D scanning strategies to generate a complete set of point clouds of physical objects through multi-view scanning and data registration [12,13,14]. Furthermore, the control of quadrupedal robots has seen experimental success in achieving robust and agile locomotion, in the 3D space [15,16]. The utilization of representation-free model predictive control and exact feedback linearization has been implemented on quadrupedal robots, contributing to the stabilization of periodic gaits for quadrupedal locomotion [8]. Additionally, the application of hybrid dynamical systems has achieved physically effective and robust instances of all virtual bipedal gaits on quadrupedal robots [17].

Collectively, all of the above developments tend to transform robots from simple programmable machines into intelligent entities capable of collecting and analyzing complex environmental data, learning from their surroundings, making intricate decisions, and executing autonomous tasks with an unprecedented level of sophistication.

## 3. Autonomous Reality Modeling for Cultural Heritage (ARM4CH)

The ARM4CH proposed system is designed to automate the 3D Reality Modelling procedures in the field of Cultural Heritage by utilizing both aerial and ground Robotic Agents (RAs). Ground Robots (wheeled or quadrupedal) may navigate terrains with excellent levels of mobility, performing automated operations, tasks, and data capture safely, accurately and frequently. They can enter buildings or confined spaces and capture close-up images or videos at ground level. Since they are not constrained by airspace flying regulations, they can be utilized in areas where drones are not permitted. On the other hand, aerial robots/drones are used when ground scanning is impossible. Each RA (aerial or ground) is equipped with specialized hardware and software to perform autonomous navigation and sensor data acquisition.

**Figure 1:**
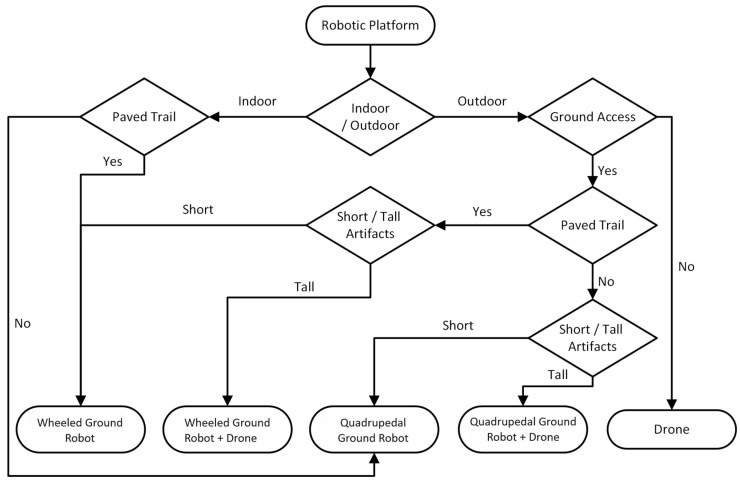
Type of RA decision flowchart.

A significant feature of ARM4CH is that both ground and aerial robotic agents may be configured to operate cooperatively. The selection of the RA (or a combination of RAs) for the survey is subject to the specifications of the CH site, such as the terrain morphology, necessary regulations to be followed, indoor or outdoor environment, as well as possible requirements set by stakeholders during the survey. For example, for an outdoor, large-scale CH site, with an unpaved trail and with tall artifacts (e.g., large-scale monuments such as the Acropolis of Athens), the best choice would be to employ quadrupedal robots, which have the capability to traverse in complex environments with high mobility, in co-operation with aerial RAs that can capture data from above, to offer an alternative perspective for areas that are inaccessible to the ground robots or when their sensors cannot adequately cover a Point of Interest (POI). Figure 1 depicts an indicative flowchart for the appropriate selection of the group of Robotic Agents for the Reality Modeling task, while a basic description of the RAs configuration is given in the next section.

### 3.1. Robotic Agent (RA) Architecture

**Figure 2:**
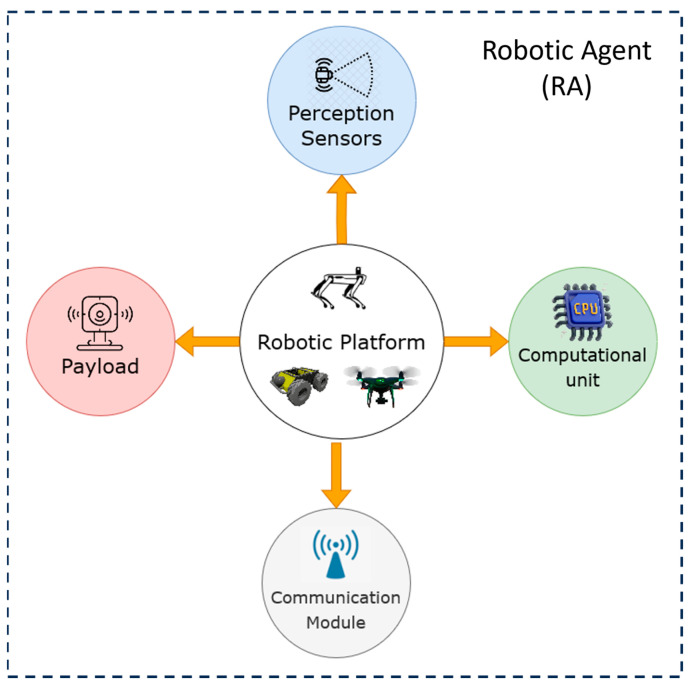
Robotic agent architecture.

As discussed earlier, the role of the RAs is to navigate autonomously in the CH site to perform the survey. In this section, we present two kinds of agents consisting of five main components (as seen in Figure 2), namely a quadrupedal robot and a drone.

**Figure 3:**
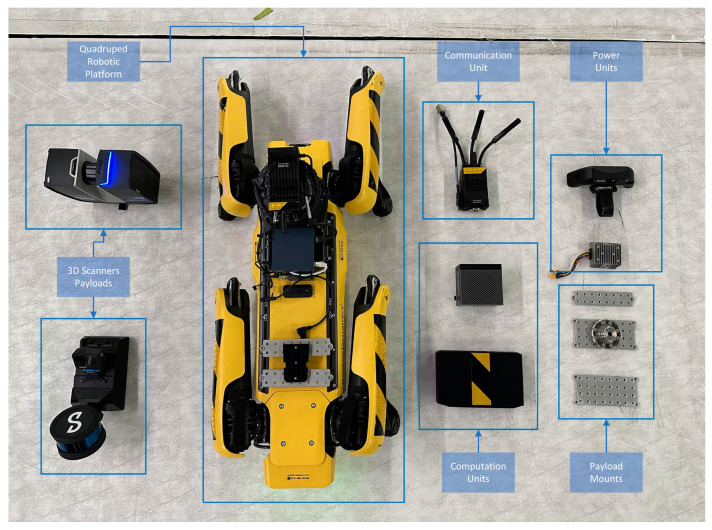
Quadruped RA components.

**Figure 4:**
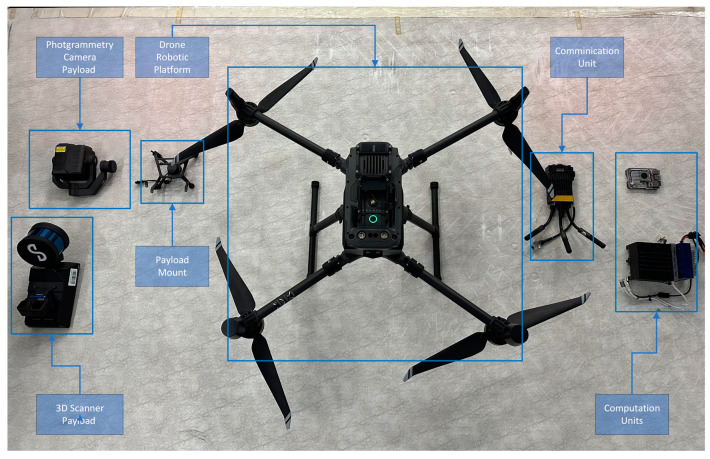
Drone RA components.

The first component is the robotic platform by itself, which is a mobile robot capable of traversing the CH environment. As already mentioned above, there are many different types of mobile robots with different abilities, advantages, and disadvantages such as wheeled robots [18], quadrupedal robots, and aerial robots/drones [19]. The platform is the main core of the mobile agent that carries all of the necessary hardware including the perception sensors, the computation unit, the communication module, and finally, the payload, which is the actual Reality Modelling sensor. The parts of the RAs for the quadrupedal and aerial robots are shown in detail in Figure 3 and Figure 4, respectively.

The perception sensors are responsible for collecting information about the state of the robotic platform and the physical environment around it. These sensors include LiDARs, RGB and depth cameras, motor encoders, Global Navigation Satellite Systems (GNSSs), and Inertial Measurement Units (IMUs), just to name the basics [19,20]. All of the collected data are managed in real time from numerous algorithms to control the robot.

Moreover, the computation unit is crucial for RA functions and operations in order to achieve onboard data processing, data collection, and management for effective navigation. This unit leverages raw data from all of the perception sensors and employs algorithms for odometry, pose estimation, Simultaneous Localization and Mapping (SLAM) [21], obstacle avoidance, motion and path planning, object detection [22], and the exploration of the environment.

The communication module is pivotal for enabling Robotic Agents to interact and co-operate [23]. It facilitates the exchange of information through protocols like TCP-IP, allowing robots to coordinate tasks, share sensor data, and make collective decisions. For instance, in the case of multiple RAs in a collaborative operation mode, it can divide tasks based on RA capabilities or current status, ensuring efficient task completion [24]. Sharing sensor inputs helps in constructing a comprehensive environmental understanding, enhancing decision-making. Additionally, communication is essential for monitoring the system’s process by human supervisors.

Finally, the payload is the main instrument dedicated to the collection of survey data, which in our case is the 3D representation of a CH site. The type of 3D sensor may vary depending on the requirements of the scan, the desired 3D point cloud resolution and accuracy [25,26] and the nature of the artifacts (e.g., shape, size, material, etc.) [27]. Possible payloads may be selected from a group of sensors like 3D Terrestrial Scanners, LiDAR sensors, depth sensors, 360 cameras, and other 2D imaging sensors (RGB or thermal cameras). Table 1 presents a brief comparison with the pros and cons of the three basic 3D point cloud acquisition methods, namely TLS, mobile scanners, and photogrammetry/SfM. In summary, TLS offer very high resolution and accuracy, typically ranging from millimeters to a few centimeters (e.g., 3.5 mm@ 25 m, 1 ΜPoint/s), while mobile/SLAM scanners offer resolutions around 2–3 cm, 5 mm@10 m, 0.5 Mpoint/s. Hence, the resolution and accuracy of the payloads attached to the RAs specify the resolution and accuracy of every ARM4CH scanning mission.

### 3.2. Methodology

**Figure 5:**
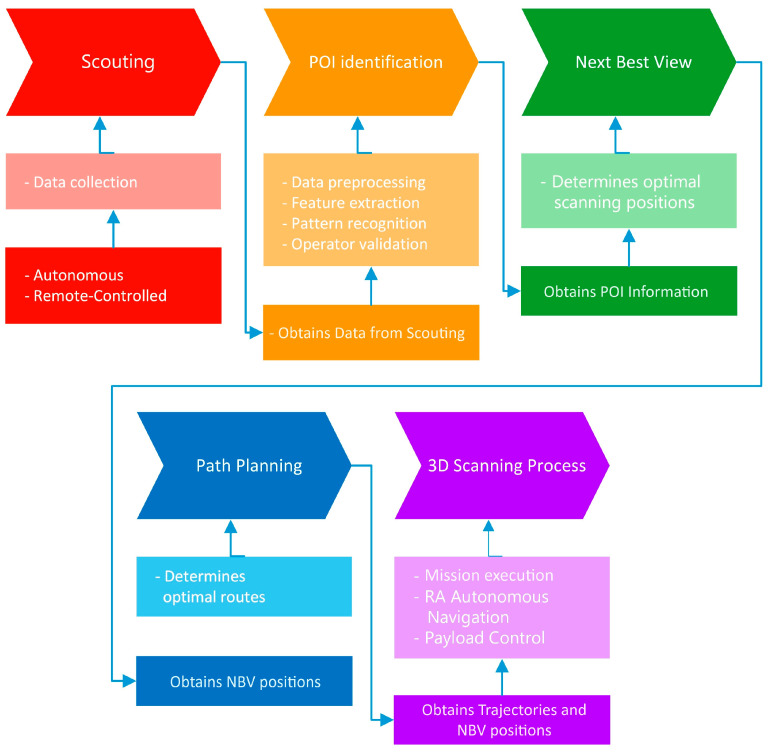
Flow diagram of the ARM4CH methodology.

The ARM4CH methodology comprises of five stages, namely scouting, Point-of-Interest (POI) identification, NBV detection, path planning, and finally, the on-site 3D scanning survey. A flow diagram of ARM4CH is highlighted in Figure 5.

#### 3.2.1. Scouting

The goal of this step is to collect information related to the heritage site that will be later used for the navigation of the robot during the autonomous scanning process at the final stage. This dynamic operation comes with the limitations of low point cloud resolution, high noise due to motion distortion, and the inability to record an RGB-mapped point cloud. However, as a great advantage, LiDAR sensors calculate rapidly a coarse 3D topological map of the surveyed area, providing occupancy maps for the execution of the next steps.

In the scouting stage, various information may be given by the operator such as general areas of exploration/responsibility and preferable routes for exploration [28], as well as locations of no-go zones (either for ground or aerial units). For successfully achieving the above tasks, fiducial markers (e.g., AprilTags, ArUco markers) [29] and geotagged site images should be incorporated. During scouting, the navigation of the robotic platform can be performed either with autonomous or remote-controlled exploration.

For the former, the robotic agent (RA) autonomously navigates all accessible pathways within a predefined area of responsibility. The primary objective is to maximize coverage of the area, while minimizing the distance traveled. The area of responsibility, along with any designated no-go zones, is the input data to a Frontier-Based Exploration algorithm [7]. This algorithm, in combination with a Simultaneous Localization and Mapping (SLAM) algorithm [21], will then generate an occupancy grid map [30] of the heritage site. For the latter (remote-controlled exploration), the RA is navigated by a remote operator, who manually controls its movement through the predefined area of responsibility [13]. While the operator directs the robot, a SLAM algorithm continuously processes sensor data to generate an occupancy grid map of the environment. This approach allows for human oversight in navigating complex or sensitive areas, while still benefiting from the automated mapping capabilities of the SLAM algorithm [31].

#### 3.2.2. Point of Interest (POI) Identification

This step involves detecting and recognizing significant locations or objects (Point of Interest—POI) within the heritage environment, which can be accomplished either manually or automatically using Machine Learning.

**Figure 6:**
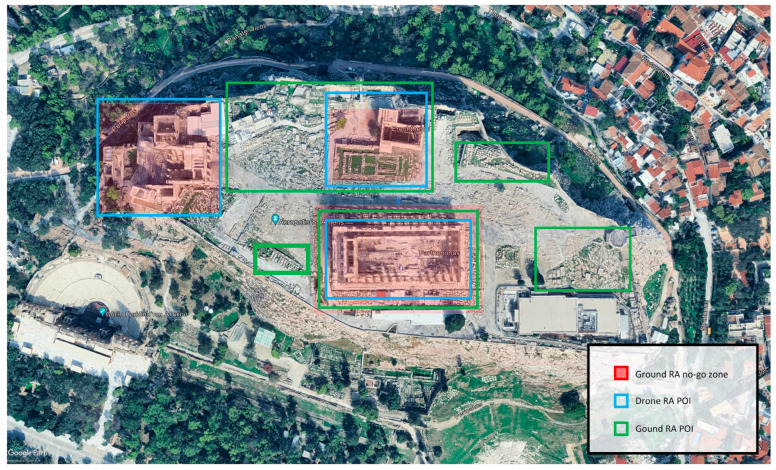
Manual POI annotations.

In manual operation, the operator selects POIs within a visualized multimodal data environment. This environment integrates and displays data collected by the robot’s sensors during scouting, including georeferenced images, 3D point clouds, and occupancy grid maps [30]. This digital representation enables the operator to effectively identify areas of interest. Figure 6 displays an example of POI selection in a georeferenced image of a CH site, where ground and drone vehicle POIs are selected along with no-go zones.

On the other hand, the automatic POI identification process is a complex Machine Learning classification task that includes several stages [32]. First, data preprocessing is performed to reduce noise. Next, feature extraction is conducted to identify key characteristics within the data [33] and finally, pattern recognition and machine learning techniques are applied to classify and cluster potential POIs [34]. The identified POIs are subsequently mapped onto an occupancy grid, allowing for precise localization and visualization. The final step in automatic POI identification involves operator validation of the identified POIs to ensure accuracy and relevance.

#### 3.2.3. Next Best View Detection

**Figure 7:**
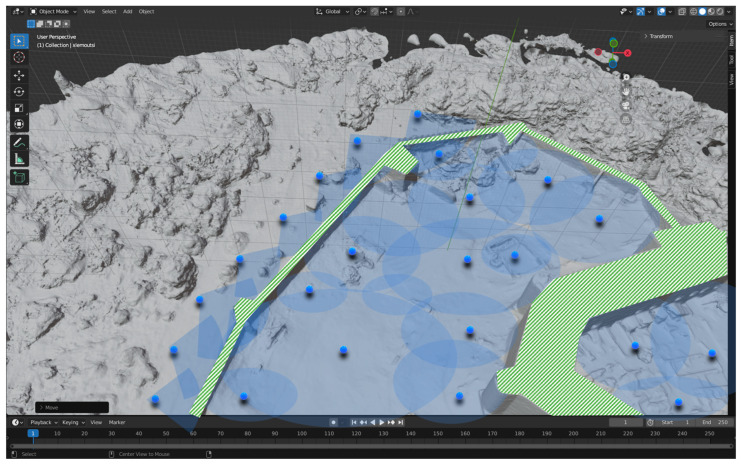
Visualization tool for the calculation of optimum positions (Next Best View) for terrestrial 3D scanning (TLS—blue dots) in Chlemoutsi castle. Uncovered parts are shown in green color. In this figure, Neu-NBV [44] was simulated in Unity and the results are shown in Blender. The 3D model was acquired from a previous manual survey.

The Next Best View (NBV) process aims to identify the optimal viewpoints for the RA to capture comprehensive 3D scans of the heritage site. Initially, the NBV algorithm evaluates the current state of the environment and determines the next best position and orientation for the robot’s sensors. The goal is to maximize the amount of new information captured, reduce redundant scanning, and ensure high-quality, complete 3D models. To this end, estimating the Next Best View (NBV) in 3D environments is a critical aspect of autonomous data acquisition and 3D reconstruction. It involves determining the most informative viewpoint for a sensor or robotic system to capture data that maximizes the information gain, while considering factors such as occlusions, completeness, and reconstruction quality. Researchers have proposed various approaches for NBV estimation, including probabilistic frameworks [35], volumetric information gain metrics [18,19,36,37], guided NBV for the 3D reconstruction of large complex structures using Unmanned Aerial Vehicles (UAVs) [38], and strategies for selecting the next best view based on ray tracing and already available BIM information [13]. Furthermore, the NBV problem has been addressed in the context of the surface reconstruction of large-scale 3D environments with multiple UAVs [38,39], and effective exploration for Micro Aerial Vehicles (MAVs) based on expected information gain [23,39]. These approaches leverage techniques such as reinforcement learning [24,40], feature tracking, reconstruction for NBV planning [25,41], and history-aware autonomous 3D exploration [26,42]. They aim to address the challenge of selecting the most informative viewpoint for 3D mesh refinement [27,43]. Therefore, NBV is a model-based approach, running within software (virtual environment) on the basis of the prior model obtained from the coarse 3D LiDAR scan of the site or an occupancy map, to defining a planning strategy for the identification of the proper scanning positions. Figure 7 displays optimum positions (blue dots) for Terrestrial Laser Scanning in the Medieval Castle of Chlemoutsi, in Ilia, Greece (https://maps.app.goo.gl/kHMmG7A1DxN8gKLp6, accessed on 1 June 2024), while in the green color are the parts of the Monument that cannot be covered by TLS due to height constraints. These areas will be surveyed using aerial vehicle (drone).

#### 3.2.4. Path Planning

**Figure 8:**
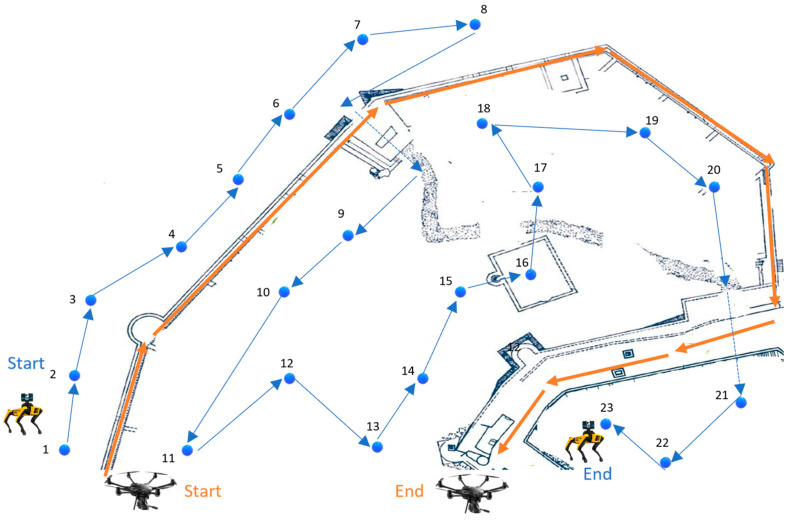
Generated paths/trajectories, with blue for the ground RA and orange arrows for the drone RA in Chlemoutsi castle. The numbers in the blue path indicate the sequence of the proposed positions (stops) for the terrestrial 3D scanning.

Path planning involves determining the optimal routes for the RA to follow, taking into account the NBV recommendations to ensure efficient and comprehensive coverage of the heritage site. Using the data collected during the scouting phase, including sparse cloud points, reference marker positions, and desirable trajectories, the path planning process creates a route that maximizes area coverage while avoiding obstacles and adhering to any specified no-go zones. Path planning algorithms, such as Rapidly-exploring random trees (RRT) and the Probabilistic Roadmap Method (PRM) [45], are employed to compute the most efficient paths. These algorithms consider the occupancy grid map [30] generated by the SLAM [46] algorithm and incorporate the NBV-determined viewpoints, ensuring that the planned paths are navigable, safe, and optimized for thorough 3D scanning. This integrated approach ensures that the robot can navigate effectively while capturing high-quality data on the heritage site. Figure 8 shows an example of the final trajectories proposed for the robot to follow during the ground and aerial surveys. At this point, Table 2 summarizes possible software packages that may be considered for the former four methodological steps (i.e., Scouting, POI, NBV, and Path Planning).

#### 3.2.5. Scanning Process

**Figure 9:**
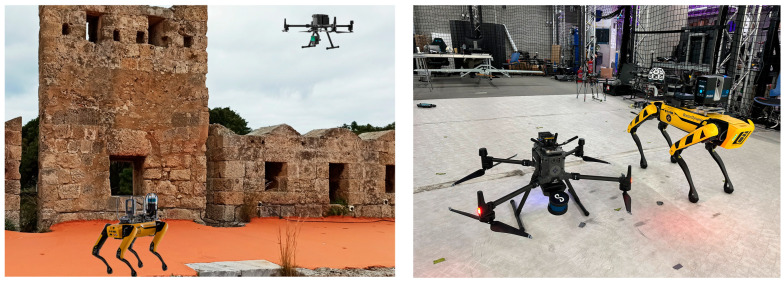
Robotic agents carrying sensors in the CH site (**left**) and in the KINESIS lab (**right**).

The final stage of the methodology is the 3D scanning process, which builds on the scouting, POI identification, NBV, and path planning stages. In this stage, the RAs follow the pre-determined optimal paths, as outlined by the path planning stage, to conduct comprehensive 3D scanning of the heritage site. Utilizing the optimal viewpoints identified during the NBV process, the RA captures high-resolution 3D data, ensuring that all significant areas and POIs are thoroughly documented. The integration of SLAM ensures continuous localization and mapping accuracy, allowing the RA to adapt in real-time to any changes or obstacles encountered. The resulting 3D scans are then compiled into detailed, high-fidelity models of the heritage site, providing a valuable resource for preservation, analysis, and further research [64]. This systematic approach guarantees that the heritage site is meticulously documented with minimal movement and maximum efficiency. Figure 9 represents a cooperative operation of an aerial and quadrupedal robotic agents (RA) in a CH site, as well as a photo with the respective RAs in the lab.

## 4. Benefits and Barriers of the ARM4CH Methodology

Using the ARM4CH methodology, researchers/surveyors may send ground/quadrupedal robots on autonomous survey missions (both indoors and outdoors) using SLAM and GPS navigation in full co-operation with aerial vehicles (UAV) for analysis, data capture, documentation, and 3D scanning. The great benefits exceed the task of Cultural Heritage 3D scanning, since cooperative autonomous Reality Modelling/inspection features the following advantages:

- The ability to schedule robots remotely on unsupervised data capture and monitoring missions, 24/7, with specific field coverage.
- Ensure accuracy by capturing data from the same locations (viewpoints) multiple times, thus making direct data comparison feasible.
- The ability to create specific schedule plans to capture up-to-date data reliably.
- Reviewing, surveying, and inspecting spaces or places of critical/specific importance or those that pose a level of danger to the human surveyor.
- Complement the advantages of various sensor technologies and boost performance.
- Continuous or periodic monitoring. Thus, once a problem is confirmed, a maintenance team may be sent.

**Figure 10:**
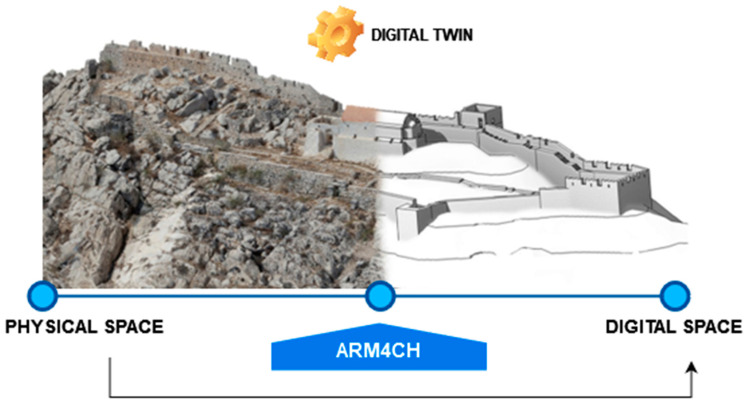
ARM4CH as a catalyst for continuous model updates in the Digital Twin concept (the case study in this example is the castle of Chalki, Dodecanese).

From the above it is evident that a great advantage of ARM4CH methodology is that it may be replicated/executed systematically, as many times as necessary in forthcoming periods, providing the ability to complete follow-up scans of the same place/site. Those follow-up scans introduce the concept of the fourth dimension (4D) in RM, since now the dimension of time is considered. Consecutive follow-up scans facilitate timeline comparison and monitoring of a constantly changing site and thus flag locations that need emergency actions in times of crisis. To this end, ARM4CH may be extremely valuable during the process of establishing and maintaining a Digital Twin (DT) of a CH site or space. This is due to the fact that, “… a DT is a virtual instance of a physical system that is continually updated with the latter’s performance” [65], leveraging the most updated available sensor data, to mirror the corresponding physical counterpart. Figure 10 demonstrates a graphical representation of a Digital Twin, in which ARM4CH may be used as a middleware to maintain updates of the site status.

To summarize the potential benefits and possible barriers of ARM4CH methodology, Table 3 analyses both advantages and disadvantages of embracing this Industry 4.0 methodological framework applied to the field of Cultural Heritage Digitization and Management in general.

## 5. Discussion and Future Work

In this paper, we briefly presented the main steps and stages for a completely new methodology (ARM4CH) to ensure autonomous 3D scanning and digitization for Cultural Heritage spaces. Key enablers of ARM4CH are the following: (a) a technology core platform comprising autonomous ground robots, as well as UAVs, that work cooperatively to navigate and survey large areas using the latest technological sensors and deep learning-based computer vision [66,67,68], and (b) the operation of a software visualization tool that resolves the Next Best View problem in 3D meshes and identifies the optimum viewpoint position and scanning path for total survey coverage by ground and aerial (drones) robotic agents.

As already mentioned in Section 4, such a methodology could be essential for a “dynamic” DT of a cultural space to actively respond to the urgent need for the efficient management, resilience, and sustainability of CH sites, facilities, buildings, structures (indoors and/or outdoors), and their surrounding environment. This undeniable need is emphasized especially in the light of climate change and the necessity of energy saving.

**Figure 11:**
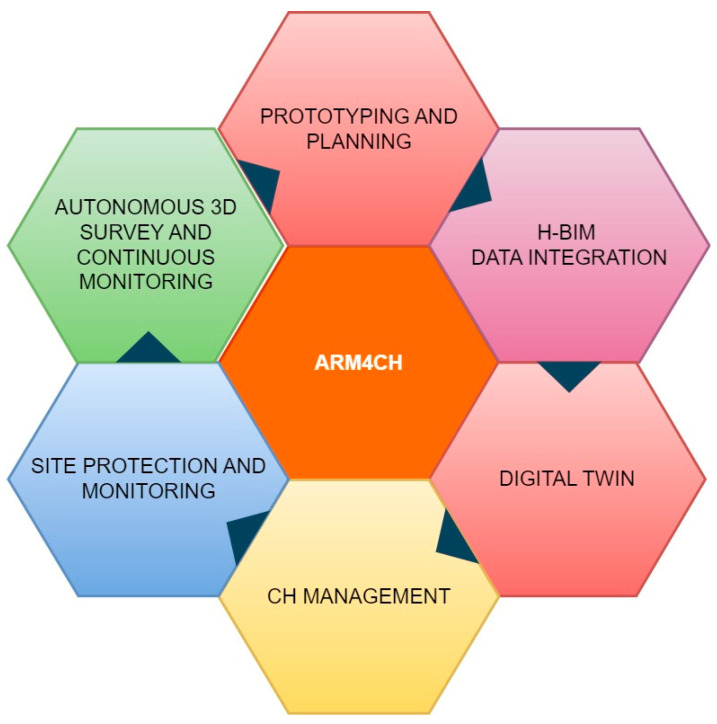
ARMCH as a core platform for various actions related to Cultural Heritage.

Therefore, since the preservation and safeguarding of our Cultural Heritage is an urgent responsibility, there is an increased requirement for automated actions and methodologies to assist the preservation, data fusion/integration, site monitoring, and holistic management of CH. Using ARM4CH, the “flower” of ARM4CH (see Figure 11) may blossom in critical areas of CH and shift the attention of professionals/experts from a curative towards a more preventive and sustainable approach for CH management.

As the ARM4CH methodology is not assessed yet, the future actions of our research focus on the provision of a proof-of-concept on an actual CH site. Hence, to validate and verify this framework in a case study, the sequence of necessary steps includes the following:

1. Comparison, evaluation and final selection of the algorithms: This will ensure the seamless operation of the equipment (RAs and payload), as well as those responsible for scouting, POI, NBV, path planning, and scanning.
2. Experimentation and training on a simulated environment: After step 1, this stage involves the training of operational RAs using the latest simulation software platforms, such as the Robot Operation System (ROS) [69], NVIDIA Omniverse [70], and Gazebo [71]. Those platforms are core modules that provide pre-trained models augmented with synthetic data to design, test, and train the autonomous navigation of RAs and deliver scalable and physically accurate virtual environments for high-fidelity simulations.
3. Experimentation in a laboratory environment: This step involves the gradual release of operation in specific scenarios into a controlled environment. Moreover, it will verify that RAs have sufficient control, communication, awareness, and perception, as well as the ability to operate and navigate in dynamic and unpredictable indoor/outdoor environments.
4. Full deployment in a real heritage site: In this final step, ARM4CH will be released and evaluated in a large-scale Cultural Heritage park that includes various infrastructure for public services, protected monuments, and archaeological sites.

## Figures and Tables

**Table 1:** Three-dimensional scanning methods comparison table [27].

Criteria	Terrestrial Laser Scanners (TLS)	SLAM-Based/Mobile Scanners	Photogrammetry/Structure from Motion (SfM)
Accuracy and Precision	High (millimeter precision)	Moderate (depends on technology)	Variable (high under optimal conditions)
Data Collection Speed	Low (requires setup and multiple stations)	Fast (on-the-go collection)	Medium (depends on the required accuracy and complexity)
Cost	High (expensive hardware)	Moderate (less expensive than Terrestrial Laser)	Low to high (depends on camera equipment)
Operational Complexity	High (requires skilled operation)	Moderate (easier in complex environments)	Moderate (requires photographic expertise)
Environmental Constraints	Sensitive to reflective surfaces	Sensitive to reflective surfaces	Highly dependent on lighting and weather conditions
Post-Processing	Intensive (cleaning, registration, and merging)	Moderate (alignment aids, needs noise reduction)	Automated but long processing and sensitive to image quality
Application Suitability	Ideal for detailed, static environments	Suitable for complex environments	Versatile for various scales under good environment conditions
Common Use Cases	Detailed architectural, archeological, and engineering documentation	Extensive and complex environments like urban areas or large buildings	Large or remote outdoor areas

**Table 2:** Potential software algorithms.

Task	Algorithm	Indicative References
Scouting	SLAM-based: ORB-based LIO-SAM ROVIO	[47,48,49]
	Exploration-based: Frontier-based Graph-based planners SOAR-based space exploration	[50,51,52]
Point of Interest	Semantic Segmentation: Segment Anything Meta (SAM) Graph-based PSPNet	[53,54,55]
	Object Detection: YOLO-based R-CNN SSD	[56,57,58]
Next Best View	Reinforcement Learning NBV (RL-NBV) Point Cloud NBV (PC-NBV) NeU-NBV	[44,59,60]
Path Planning	RRT RPM A* algorithm	[61,62,63]

**Table 3:** ARM4CH benefits and barriers.

Benefits	Detailed Analysis
Non-invasive survey and inspection	ARM4CH may carry out detailed Reality Modeling and monitoring without causing any physical disruption to the site, gathering high-resolution images, 3D scans, which are essential for a detailed analysis and documentation of the current state of the CH site.
Access to hard-to-reach areas and complex areas	Human operators are not exposed to missions and roles that might be challenging for them or to conventional surveying equipment. ARM4CH may suit perfectly for the survey or modeling of deteriorating structures of a monument.
Reduce labor costs and survey time	As CH sites often have complex architectures or difficult-to-access areas, ARM4CH may reduce the laborious and time-consuming process of 3D scanning by a human operator. The user now has a supervisory role to extensive surveys or inspections, which can be both time-consuming and expensive.
Versatility and precision	ARM4CH can be equipped with different sensors and tools, such as cameras, thermal imaging, and LIDAR, allowing it to perform a wide range of monitoring and surveying tasks with high precision, reducing the chances of human error and ensuring high-quality data collection.
Consistency and optimization	ARM4CH may perform tasks autonomously and systematically, following predefined routes and schedules, ensuring optimized, consistent, and reliable data collection with high precision and consistency.
Survey replication	ARM4CH may be replicated/executed systematically, as many times as necessary, providing the ability to complete follow-up scans of the CH site and update its Digital Twin or Heritage Building Information Model (H-BIM).
Regular monitoring	ARM4CH may lead to regular and consistent monitoring, providing up-to-date information on the CH site (i.e., detecting gradual changes or deterioration over time, damage, structural weaknesses, etc.), facilitating an immediate response to potential problems.
Long-term preservation and management	The detailed and systematic data collected by ARM4CH may assist curators, and conservation experts in prototype planning and executing restoration projects and a holistic CH management strategy.
Cost	The initial cost for creating the ARM4CH core platform including RAs and sensors is high, which might be a barrier for surveying companies or CH stakeholders that would like to operate this methodology.
Training and expertise transfer	The execution of ARM4CH and data management requires the presence of an expert in the survey team, who would supervise the process. This may necessitate additional resources for staff training or hiring skilled personnel.
Data management	If regular surveys are needed, a robust data infrastructure should be available, since large volumes of data collected need to be stored, processed, and managed (e.g., a complete DT platform).
Ethical and cultural concerns	The use of Industry 4.0 equipment in CH sites might raise ethical or cultural concerns among stakeholders who prefer traditional methods or have concerns about the use of frameworks of the latest technology (i.e., malfunctions that may cause unintentional damage to the site).

## Data Availability

The original contributions presented in the study are included in the article, further inquiries can be directed to the corresponding author.

## References

[1] Hu D., Minner J. (2023). UAVs and 3D City Modeling to Aid Urban Planning and Historic Preservation: A Systematic Review. Remote Sens..

[2] Li Y., Zhao L., Chen Y., Zhang N., Fan H., Zhang Z. (2023). 3D LiDAR and multi-technology collaboration for preservation of built heritage in China: A review. Int. J. Appl. Earth Obs. Geoinf..

[3] Mitric J., Radulovic I., Popovic T., Scekic Z., Tinaj S. AI and Computer Vision in Cultural Heritage Preservation. Proceedings of the 2024 28th International Conference on Information Technology (IT).

[4] Caron G., Bellon O.R.P., Shimshoni I. (2023). Computer Vision and Robotics for Cultural Heritage: Theory and Applications. J. Imaging.

[5] Aicardi I., Chiabrando F., Lingua A.M., Noardo F. (2018). Recent trends in cultural heritage 3D survey: The photogrammetric computer vision approach. J. Cult. Herit..

[6] Mahmood S., Majid Z., Idris K.M. (2020). Terrestrial LiDAR sensor modeling towards optimal scan location and spatial density planning for 3D surveying. Appl. Geomat..

[7] Prieto S.A., Giakoumidis N., García De Soto B. (2024). Multiagent robotic systems and exploration algorithms: Applications for data collection in construction sites. J. Field Robot..

[8] Fawcett R.T., Pandala A., Ames A.D., Hamed K.A. (2022). Robust Stabilization of Periodic Gaits for Quadrupedal Locomotion via QP-Based Virtual Constraint Controllers. IEEE Control. Syst. Lett..

[9] Lee J., Hwangbo J., Wellhausen L., Koltun V., Hutter M. (2020). Learning quadrupedal locomotion over challenging terrain. Sci. Robot..

[10] Soori M., Arezoo B., Dastres R. (2023). Artificial intelligence, machine learning and deep learning in advanced robotics, a review. Cogn. Robot..

[11] Mikołajczyk T., Mikołajewski D., Kłodowski A., Łukaszewicz A., Mikołajewska E., Paczkowski T., Skornia M. (2023). Energy Sources of Mobile Robot Power Systems: A Systematic Review and Comparison of Efficiency. Appl. Sci..

[12] Chen L., Hoang D., Lin H., Nguyen T. (2016). Innovative methodology for multi-view point cloud registration in robotic 3d object scanning and reconstruction. Appl. Sci..

[13] Park S., Yoon S., Ju S., Heo J. (2023). BIM-based scan planning for scanning with a quadruped walking robot. Autom. Constr..

[14] Kim P., Park J., Cho Y. As-is geometric data collection and 3D visualization through the collaboration between UAV and UGV. Proceedings of the International Symposium on Automation and Robotics in Construction (ISARC).

[15] Peers C., Motawei M., Richardson R., Zhou C. Development of a teleoperative quadrupedal manipulator. Proceedings of the UKRAS21 Conference: Robotics at Home Proceedings.

[16] Ding Y., Pandala A., Li C., Shin Y., Park H.W. (2021). Representation-free model predictive control for dynamic motions in quadrupeds. IEEE Trans. Robot..

[17] Hutter M., Gehring C., Jud D., Lauber A., Bellicoso C.D., Tsounis V., Hwangbo J., Bodie K., Fankhauser P., Bloesch M. ANYmal—A highly mobile and dynamic quadrupedal robot. Proceedings of the 2016 IEEE/RSJ International Conference on Intelligent Robots and Systems (IROS).

[18] Borkar K.K., Aljrees T., Pandey S.K., Kumar A., Singh M.K., Sinha A., Sharma V. (2023). Stability Analysis and Navigational Techniques of Wheeled Mobile Robot: A Review. Processes.

[19] Rubio F., Valero F., Llopis-Albert C. (2019). A review of mobile robots: Concepts, methods, theoretical framework, and applications. Int. J. Adv. Robot. Syst..

[20] Camurri M., Ramezani M., Nobili S., Fallon M. (2020). Pronto: A Multi-Sensor State Estimator for Legged Robots in Real-World Scenarios. Front. Robot. AI.

[21] Macario Barros A., Michel M., Moline Y., Corre G., Carrel F. (2022). A Comprehensive Survey of Visual SLAM Algorithms. Robotics.

[22] Mittal S. (2019). A Survey on optimized implementation of deep learning models on the NVIDIA Jetson platform. J. Syst. Archit..

[23] Li Y., Du S., Kim Y. Robot swarm MANET cooperation based on mobile agent. Proceedings of the 2009 IEEE International Conference on Robotics and Biomimetics (ROBIO).

[24] Ivanov M., Sergiyenko O., Tyrsa V., Lindner L., Reyes-García M., Rodríguez-Quiñonez J.C., Hernández-Balbuena D. (2020). Data Exchange and Task of Navigation for Robotic Group.

[25] Kalvoda P., Nosek J., Kuruc M., Volarik T., Kalvodova P. (2020). Accuracy Evaluation and Comparison of Mobile Laser Scanning and Mobile Photogrammetry Data. IOP Conf. Ser. Earth Environ. Sci..

[26] Dering G.M., Micklethwaite S., Thiele S.T., Vollgger S.A., Cruden A.R. (2019). Review of drones, photogrammetry and emerging sensor technology for the study of dykes: Best practises and future potential. J. Volcanol. Geotherm. Res..

[27] Daneshmand M., Helmi A., Avots E., Noroozi F., Alisinanoglu F., Arslan H.S., Gorbova J., Haamer R.E., Ozcinar C., Anbarjafari G. 3D Scanning: A Comprehensive Survey. Proceedings of the Conference on Computer Vision and Pattern Recognition.

[28] Chen K., Reichard G., Akanmu A., Xu X. (2021). Geo-registering UAV-captured close-range images to GIS-based spatial model for building façade inspections. Autom. Constr..

[29] Kalaitzakis M., Cain B., Carroll S., Ambrosi A., Whitehead C., Vitzilaios N. (2021). Fiducial Markers for Pose Estimation. J. Intell. Robot. Syst..

[30] Hornung A., Wurm K.M., Bennewitz M., Stachniss C., Burgard W. (2013). OctoMap: An efficient probabilistic 3D mapping framework based on octrees. Auton. Robot..

[31] Wallace D., He Y.H., Vaz J.C., Georgescu L., Oh P.Y. Multimodal Teleoperation of Heterogeneous Robots within a Construction Environment. Proceedings of the 2020 IEEE/RSJ International Conference on Intelligent Robots and Systems (IROS).

[32] Pierdicca R., Paolanti M., Matrone F., Martini M., Morbidoni C., Malinverni E.S., Lingua A.M. (2020). Point Cloud Semantic Segmentation Using a Deep Learning Framework for Cultural Heritage. Remote Sens..

[33] Câmara A., de Almeida A., Caçador D., Oliveira J. (2023). Automated methods for image detection of cultural heritage: Overviews and perspectives. Archaeol. Prospect..

[34] Fiorucci M., Verschoof-Van Der Vaart W.B., Soleni P., Le Saux B., Traviglia A. (2022). Deep Learning for Archaeological Object Detection on LiDAR: New Evaluation Measures and Insights. Remote Sens..

[35] Potthast C., Sukhatme G.S. (2014). A probabilistic framework for next best view estimation in a cluttered environment. J. Vis. Commun. Image Represent..

[36] Bircher A., Kamel M., Alexis K., Oleynikova H., Siegwart R. (2018). Receding horizon path planning for 3D exploration and surface inspection. Auton. Robot..

[37] Delmerico J., Isler S., Sabzevari R., Scaramuzza D. (2018). A comparison of volumetric information gain metrics for active 3D object reconstruction. Auton. Robot..

[38] Almadhoun R., Abduldayem A., Taha T., Seneviratne L., Zweiri Y. (2019). Guided Next Best View for 3D Reconstruction of Large Complex Structures. Remote Sens..

[39] Palazzolo E., Stachniss C. (2018). Effective Exploration for MAVs Based on the Expected Information Gain. Drones.

[40] Kaba M.D., Uzunbas M.G., Lim S.N. A Reinforcement Learning Approach to the View Planning Problem. Proceedings of the IEEE Conference on Computer Vision and Pattern Recognition (CVPR).

[41] Trummer M., Munkelt C., Denzler J. (2009). Combined GKLT Feature Tracking and Reconstruction for Next Best View Planning.

[42] Wang Y., Del Bue A. (2020). Where to Explore Next? ExHistCNN for History-Aware Autonomous 3D Exploration.

[43] Morreale L., Romanoni A., Matteucci M. (2019). Predicting the Next Best View for 3D Mesh Refinement.

[44] Jin L., Chen X., Rückin J., Popović M. NeU-NBV: Next Best View Planning Using Uncertainty Estimation in Image-Based Neural Rendering. Proceedings of the 2023 IEEE/RSJ International Conference on Intelligent Robots and Systems (IROS).

[45] Muhammad A., Abdullah N.R.H., Ali M.A., Shanono I.H., Samad R. Simulation Performance Comparison of A*, GLS, RRT and PRM Path Planning Algorithms. Proceedings of the 2022 IEEE 12th Symposium on Computer Applications & Industrial Electronics (ISCAIE).

[46] Bujanca M., Shi X., Spear M., Zhao P., Lennox B., Luján M. Robust SLAM Systems: Are We There Yet?. Proceedings of the 2021 IEEE/RSJ International Conference on Intelligent Robots and Systems (IROS).

[47] Campos C., Elvira R., Rodríguez JJ G., Montiel J.M., Tardós J.D. (2021). ORB-SLAM3: An Accurate Open-Source Library for Visual, Visual–Inertial, and Multimap SLAM. IEEE Trans. Robot..

[48] Shan T., Englot B., Meyers D., Wang W., Ratti C., Rus D. LIO-SAM: Tightly-coupled Lidar Inertial Odometry via Smoothing and Mapping. Proceedings of the 2020 IEEE/RSJ International Conference on Intelligent Robots and Systems (IROS).

[49] Bloesch M., Omari S., Hutter M., Siegwart R. Robust visual inertial odometry using a direct EKF-based approach. Proceedings of the 2015 IEEE/RSJ International Conference on Intelligent Robots and Systems (IROS).

[50] Yamauchi B. A frontier-based approach for autonomous exploration. In Proceedings 1997 IEEE International Symposium on Computational Intelligence in Robotics and Automation CIRA’97. “Towards New Computational Principles for Robotics and Automation”.

[51] Dang T., Tranzatto M., Khattak S., Mascarich F., Alexis K., Hutter M. (2020). Graph-based subterranean exploration path planning using aerial and legged robots, Special Issue on Field and Service Robotics (FSR). J. Field Robot..

[52] Luo F., Zhou Q., Fuentes J., Ding W., Gu C. (2022). A Soar-Based Space Exploration Algorithm for Mobile Robots. Entropy.

[53] Segment Anything. https://segment-anything.com/.

[54] Felzenszwalb P.F., Huttenlocher D.P. (2004). Efficient Graph-Based Image Segmentation. Int. J. Comput. Vis..

[55] Zhao H., Shi J., Qi X., Wang X., Jia J. Pyramid Scene Parsing Network. Proceedings of the 2017 IEEE Conference on Computer Vision and Pattern Recognition (CVPR).

[56] Redmon J., Divvala S., Girshick R., Farhadi A. You Only Look Once: Unified, Real-Time Object Detection. Proceedings of the 2016 IEEE Conference on Computer Vision and Pattern Recognition (CVPR).

[57] Girshick R., Donahue J., Darrell T., Malik J. Rich Feature Hierarchies for Accurate Object Detection and Semantic Segmentation. Proceedings of the 2014 IEEE Conference on Computer Vision and Pattern Recognition.

[58] Liu W., Anguelov D., Erhan D., Szegedy C., Reed S., Fu C.Y., Berg A.C., Leibe B., Matas J., Sebe N., Welling M. (2016). SSD: Single Shot MultiBox Detector. Computer Vision—ECCV 2016. ECCV 2016. Lecture Notes in Computer Science.

[59] Wang T., Xi W., Cheng Y., Han H., Yang Y. (2024). RL-NBV: A deep reinforcement learning based next-best-view method for unknown object reconstruction. Pattern Recognit. Lett..

[60] Zeng R., Zhao W., Liu Y.-J. PC-NBV: A Point Cloud Based Deep Network for Efficient Next Best View Planning. Proceedings of the IEEE/RSJ International Conference on Intelligent Robots and Systems (IROS).

[61] Moon C.B., Chung W. (2015). Kinodynamic Planner Dual-Tree RRT (DT-RRT) for Two-Wheeled Mobile Robots Using the Rapidly Exploring Random Tree. IEEE Trans. Ind. Electron..

[62] Kavraki L.E., Svestka P., Latombe J.C., Overmars M.H. (1996). Probabilistic roadmaps for path planning in high-dimensional configuration spaces. IEEE Trans. Robot. Autom..

[63] Hart P.E., Nilsson N.J., Raphael B. (1968). A Formal Basis for the Heuristic Determination of Minimum Cost Paths. IEEE Trans. Syst. Sci. Cybern..

[64] Parrinello S., Picchio F. (2023). Digital Strategies to Enhance Cultural Heritage Routes: From Integrated Survey to Digital Twins of Different European Architectural Scenarios. Drones.

[65] Cimino C., Ferretti G., Leva A. (2021). Harmonising and integrating the digital twins multiverse: A paradigm and a toolset proposal. Comput. Ind..

[66] Osco L.P., Junior J.M., Ramos A.P.M., de Castro Jorge L.A., Fatholahi S.N., de Andrade Silva J., Matsubara E.T., Pistori H., Gonçalves W.N., Li J. (2021). A review on deep learning in UAV remote sensing. Int. J. Appl. Earth Obs. Geoinf..

[67] Qiu Q., Lau D. (2023). Real-time detection of cracks in tiled sidewalks using YOLO-based method applied to unmanned aerial vehicle (UAV) images. Autom. Constr..

[68] Mittal P., Singh R., Sharma A. (2020). Deep learning-based object detection in low-altitude UAV datasets: A survey. Image Vis. Comput..

[69] Robot Operation System (ROS). https://www.ros.org/.

[70] NVIDIA Omniverse. https://www.nvidia.com/en-eu/omniverse/.

[71] Gazebo. https://gazebosim.org.

